# Comparison of personal exposure to black carbon levels with fixed-site monitoring data and with dispersion modelling and the influence of activity patterns and environment

**DOI:** 10.1038/s41370-024-00653-2

**Published:** 2024-02-22

**Authors:** Olena Gruzieva, Antonios Georgelis, Niklas Andersson, Christer Johansson, Tom Bellander, Anne-Sophie Merritt

**Affiliations:** 1 Institute of Environmental Medicine, Karolinska Institutet, Stockholm, Sweden; 2 Centre for Occupational and Environmental Medicine, Region Stockholm, Stockholm, Sweden; 3 Department of Environmental Science, Stockholm University, Stockholm, Sweden; 4 Environment and Health Administration, SLB-analys, Stockholm, Sweden

**Keywords:** Black carbon, Personal exposure, Fixed-site monitoring, Dispersion modelling, Time-activity pattern

## Abstract

**Background:**

Short-term studies of health effects from ambient air pollution usually rely on fixed site monitoring data or spatio-temporal models for exposure characterization, but the relation to personal exposure is often not known.

**Objective:**

We aimed to explore this relation for black carbon (BC) in central Stockholm.

**Methods:**

Families (*n* = 46) with an infant, one parent working and one parent on parental leave, carried battery-operated BC instruments for 7 days. Routine BC monitoring data were obtained from rural background (RB) and urban background (UB) sites. Outdoor levels of BC at home and work were estimated in 24 h periods by dispersion modelling based on hourly real-time meteorological data, and statistical meteorological data representing annual mean conditions. Global radiation, air pressure, precipitation, temperature, and wind speed data were obtained from the UB station. All families lived in the city centre, within 4 km of the UB station.

**Results:**

The average level of 24 h personal BC was 425 (s.d. 181) ng/m^3^ for parents on leave, and 394 (s.d. 143) ng/m^3^ for working parents. The corresponding fixed-site monitoring observations were 148 (s.d. 139) at RB and 317 (s.d. 149) ng/m^3^ at UB. Modelled BC levels at home and at work were 493 (s.d. 228) and 331 (s.d. 173) ng/m^3^, respectively. UB, RB and air pressure explained only 21% of personal 24 h BC variability for parents on leave and 25% for working parents. Modelled home BC and observed air pressure explained 23% of personal BC, and adding modelled BC at work increased the explanation to 34% for the working parents.

**Impact:**

Short-term studies of health effects from ambient air pollution usually rely on fixed site monitoring data or spatio-temporal models for exposure characterization, but the relation to actual personal exposure is often not known. In this study we showed that both routine monitoring and modelled data explained less than 35% of variability in personal black carbon exposure. Hence, short-term health effects studies based on fixed site monitoring or spatio-temporal modelling are likely to be underpowered and subject to bias.

## Introduction

Ambient air pollution has been recognized as one of the major public health concerns worldwide. Many previous studies reported associations between exposure to air pollution and a wide range of adverse health effects [1]. It is particularly alarming that the negative health effects are observed even at levels well below international air pollution standards. In the new Air Quality Guidelines, WHO recommends systematic measurements of black carbon (BC) and exposure assessments [2]. Exposure to BC is largely a measure of primary fine particles related to combustion of fossil fuel or biomass and has increasingly been used in studies of the impact of air pollution on health [3]. Epidemiological studies often estimate personal exposure using concentrations measured at fixed monitoring stations or by various modelling methods to estimate outdoor levels at place of residency. People are however exposed to different levels also depending on factors like housing and ventilation characteristics as well as on individual activity patterns. Therefore, increasing the knowledge on personal exposure to BC through a better understanding and quantifying personal exposure can contribute to a more accurate exposure assessment, and provide estimates of the level of misclassification of exposure. This is of crucial importance for evaluating the health consequences of pollutants that exhibit high temporal and spatial variability, e.g. traffic-related air pollutants.

Most existing studies investigating personal exposure to BC focused on microenvironments and activities [4–7], while other predictors have been less studied. The main aims of the present study were to quantify the short-term agreement of personal exposure measurements with observed BC levels at fixed-site monitors, and the long-term agreement with dispersion-model estimates of BC levels at the residential and work addresses, as well as exploring the effect of employment status and other possible predictors of personal exposure to BC.

## Materials and methods

### Study population

The present study was based on an ongoing cohort EMIL (Aetiological Mechanisms of air pollution effects in the Infant Lung) comprising families of about 100 newborn children from Stockholm that had been recruited after identification in a population register. Details on study design and enrolment procedures have been described elsewhere [8]. When the children were approximately one year old, parents received an invitation to participate in personal measurements of BC. Sampling took place during April 2016–June 2017.

### Black carbon exposure assessment

**Fig. 1:**
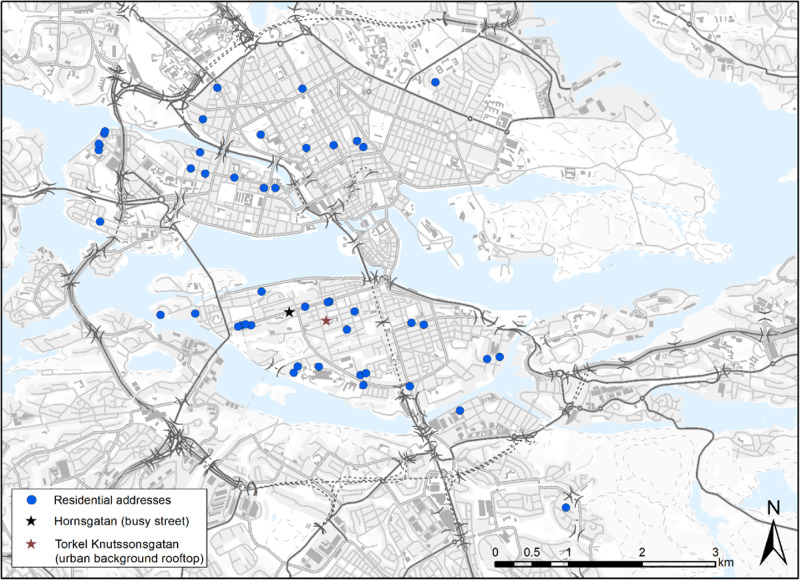
Geographical location of residential addresses of the participants in the EMIL study as well as air quality monitoring stations in Stockholm, Sweden. Each circle represents approximate position for individual residential address; stars represent air quality monitoring stations.

As many as 46 families were selected based on the criteria that one parent should be on parental leave and one parent should be working. All families resided in multistorey buildings close to the city centre, within 4 km of the urban background monitoring station (Fig. 1).

#### Personal measurements

Both parents performed measurements of BC continuously for seven consecutive days. A battery-operated MicroAeth Model AE51 (Aethlabs, San Francisco, California, US) was used with a 5-min time resolution, as has been described previously [9]. When both parents were at home, they were instructed to place the two instruments next to each other in the living room. When away from home, they were instructed to carry the instrument with the inlet positioned close to the breathing zone. At work, they were instructed to leave the instrument at the desk and bring the instrument if leaving the workplace during the day. If work was of different character than office work, they were instructed to carry the instrument unless it would be an obstacle. The filter was replaced by the participants every two days to prevent saturation and subsequent measurement bias. Mostly 2, but sometimes 3 families performed measurements in parallel.

#### Fixed-site monitoring and dispersion modelling

Rural background (RB), urban background (UB), and busy street (ST) levels of BC were monitored with automated fixed-site monitors (AETHALOMETER model AE33) by the urban air quality monitoring network of the Environment and Health Administration, SLB, Stockholm [10]. The measurement sites were: Aspvreten (RB; 80 km SW of Stockholm City), Torkel Knutssonsgatan (UB; rooftop) and Hornsgatan (ST; sidewalk). The location of the automated air quality measurement sites is shown in Fig. 1. The fixed-site BC levels were retrieved for the periods corresponding to the personal measurements. In addition, UB measurements of global radiation, pressure, precipitation, temperature, wind speed data were collected from the UB station.

We also estimated outdoor BC levels at residential and work addresses based on air quality dispersion modelling, using emission data and weather data, described in detail elsewhere [8, 11]. The modelling system is part of the Airviro Air Quality Management System (http://airviro.com) and has provided exposure estimates for several epidemiological studies and health impact assessment studies [12–14]. Briefly, residential and work addresses of families were geocoded, and the concentrations of BC were calculated in a Gaussian dispersion model using real-time meteorological data (for 1-, 24-h, and 1-week averages) or climatology (to obtain annual averages). Meteorological data (wind speed and direction, temperature gradient and horizontal and vertical wind fluctuations) were obtained from measurements in a 50 m high mast in southern Stockholm. Annual outdoor residential and work BC levels were modelled using the climatology, which consist of 360 different meteorological situations based on 15 years of meteorological measurements. The validity of using the climatology with 360 cases, rather than information from all 8760 h of a calendar year has been shown in Segersson et al. [11] and Eneroth et al. [15].

### Self-administered time-activity data

During the one-week measurement period all study participants (both parents) filled in a time-activity diary with each day divided in 4 h time intervals during the daytime and 8 h interval (22:00–06:00) during the nighttime, for which they had to provide information about their activities and locations. In brief, the participants stated how many hours and minutes they had been in a specific situation (indoors, in traffic, park, other indoor environment) during a time interval. In addition, they took notes of specific activities that could influence BC levels, such as smoking, stove use etc.

### Statistical analyses

Measurement data were checked for missing values, for anomalies (e.g. unreasonably high readings, or significant variation from the specified flow rates) as well as for any other errors (e.g. missing data due to instrument malfunction). Measurements recorded at the time of filter change along with one preceding and following record (6.7%) or an error code (2.4%) were thus excluded. Negative measurements were included in the analysis, because the integrative character of the sensor output compensates for these in the following readings [16, 17]. Cleaned 5-min data were then averaged to yield mean 24-h and 1-week values. Averages were considered valid if ≥75% of the 5-min values within each exposure window were available. Based on this criterium, a total of 38 persons on leave and 38 working persons with valid 1-week average BC values were included in the final analyses. Unreasonably high BC values were defined based on the visual inspection of the distribution of measured levels (Supplementary Fig. 1). All but 7 observed 1-h BC values were below 13,209 ng/m^3^, while 7 observations, as above 30,000 ng/m^3^ (0.03%), and therefore considered as unreasonably high. For 24-h averages, corresponding cut-off was set at 1200 ng/m^3^, which led to exclusion of 11 observations (2.2%). We also performed a sensitivity analysis including unreasonably high BC values to evaluate their potential impact on the results. Measurement season was categorized into summer (April–September) and winter (October–March). Automatic station BC data had missing values and for some analyses we imputed missing hourly values using linear regression with same-station nitrogen oxides (NOx) and particulate matter <2.5 μm in aerodynamic diameter (PM_2.5_) observations. We used concurrent and annual BC averages measured at the UB station to standardize the observed 1-week personal BC to reflect annual averages [18], according to the equation: Week averaged measured personal BC level - Week averaged measured outdoor BC at UB + Annual measured outdoor BC at UB.

The relationship between averaged measured BC concentrations and predicting variables was investigated by means of pairwise Pearson squared correlation test, as well as univariate and multiple linear regression analyses. In the latter analysis all significant variables were included in stepwise procedure. STATA Release 16.0 (StataCorp, College Station, Tex) was used for database management and statistical analyses.

## Results

### Descriptive statistics

The 1-week personal exposure to BC was on average 425 (s.d. 181) ng/m^3^ for parents on leave (*n* = 38), and 394 (s.d. 143) ng/m^3^ for working parents (*n* = 38; Supplementary Table 1). The corresponding fixed-site monitoring BC levels were 176 (s.d. 126) at RB, 319 (s.d. 75) at UB, and 1026 (s.d. 147) at ST. Modelled BC outside at home and at work were 461 (s.d. 174) and 392 (s.d. 171) ng/m^3^, respectively. Compared to exposure metrics commonly used in epidemiology, the exposure level for the parents on leave thus was on average estimated to 133% of the concentration measured in UB, and to 92% of the modelled outdoors concentration at place of residency. For the working parents the corresponding estimates were 124%, and 85%, respectively. Personal BC tended to be higher during winter than during summer, for both parents on leave and working parents (Supplementary Fig. 2).

**Fig. 2:**
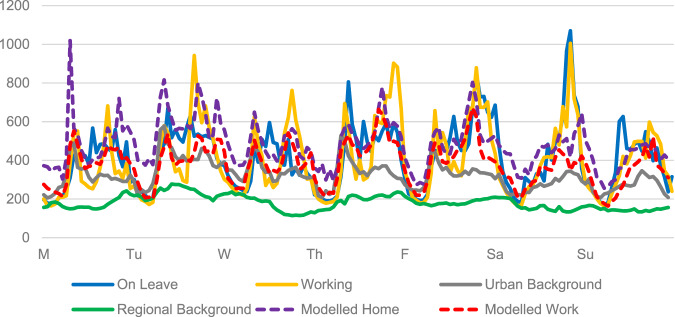
Personal (parents on leave or working), monitored (urban or regional background) and modelled BC concentrations (ng/m^3^); hourly averaged over weekdays. The vertical axis represents BC concentration (ng/m^3^). The horizontal axis represents weekdays.

In the average daily (Monday through Sunday) time series (by hour) of observed personal BC measurements, levels were clearly higher during daytime than during night-time, as were observed levels at UB (Fig. 2). At night personal UB levels were comparable to those of the RB. The levels at the RB site did not show any diurnal pattern. Looking further at the diurnal patterns, working parents showed two quite distinct peaks of elevated BC during the weekdays, corresponding to hours of commuting to and from work (Fig. 2). The morning personal peak coincided approximately in time with the peaks observed at UB, and tended to be higher than those. The observed personal afternoon peak was higher than the morning peak for all weekdays, and corresponded less to the pattern at the UB site, that showed a lower and less distinct peak compared to the morning. Also, during the central hours of the weekdays, working parents showed lower levels, indicating low indoor levels at the workplace.

The pattern during the workdays was less distinct for the parents on leave, but the levels were higher than the UB levels, especially in the afternoon. Saturdays both parents experienced a strong and virtually identical afternoon peak, while on Sundays the parent on leave tended to have an increased level in the morning, and the working parent in the afternoon. The weekday patterns of modelled (1 h mean) outdoor levels at home and at work coincided only occasionally with the observed personal levels (Fig. 2).

According to the self-administered diary, the distribution of time spent in different micro-environments was quite similar for both parents, with the expected exception for time spent at work for the working parent (and similarly less at home; Supplementary Table S2).

### Correlation of BC exposure measurements

In the short-term perspective (24 h average) the parents’ personal BC levels moderately correlated (coefficient of determination R^2^ = 0.48) with each other (Table 1A). As much as 12% of variance in measured BC levels of parents on a leave and 15% of variance among working parents could be explained by modelled outdoor levels at the home, and 14-19% with observed levels at RB and UB, while less than 1% of variance in both parents could be explained by ST levels. Modelled outdoor levels at work explained 37% of variance in BC levels measured in working parent. Among the weather variables only air pressure showed low correlation, explaining 3% of personal BC levels (R^2^ = 0.03 for both parents). Time spent in traffic or in other microenvironments did not correlate with observed personal levels (data not shown).

**Table 1:** Pairwise correlation matrix of black carbon levels (Coefficients of determination (R2)).

A) BC averaged over 24 h												
	Personal measurements		Modelled for home address	Modelled for work address	Measured at continuous monitor							
Personal measurements:	Parent on a leave	Working parent			Urban background	Rural background	Street level	Global radiation	Air pressure	Precipitation	Temperature	Wind speed
Parent on a leave	1.0											
Working parent	0.48	1.0										
Modelled for home address	0.12	0.15	1.0									
Modelled for work address	NA	0.37	0.55	1.0								
Measured at continuous monitor:												
Urban background	0.14	0.16	0.46	0.46	1.0							
Rural background	0.18	0.19	0.48	0.67	0.53	1.0						
Street level	0.004	0.004	0.15	0.05	0.27	0.05	1.0					
Global radiation	0.07	0.03	0.005	0.02	0.01	0.06	0.04	1.0				
Air pressure	0.03	0.03	0.02	0.04	0.04	0.006	0.008	0.01	1.0			
Precipitation	0.004	0.004	0.001	0.0009	0.005	0.003	0.002	0.02	0.05	1.0		
Temperature	0.01	0.002	0.11	0.006	0.01	0.0004	0.07	0.42	0.03	0.007	1.0	
Wind speed	0.002	0.004	0.13	0.04	0.10	0.0009	0.14	0.04	0.07	0.01	0.05	1.0
**B) BC averaged over 1 week**												
	**Personal measurements**		**Modelled for home address**	**Modelled for work address**	**Measured at continuous monitor**							
Personal measurements:	Parent on a leave	Working parent			Urban background	Rural background	Street level	Global radiation	Air pressure	Precipitation	Temperature	Wind speed
Parent on a leave	1.0											
Working parent	0.69	1.0										
Modelled for home address	0.08	0.09	1.0									
Modelled for work address	NA	0.45	0.26	1.0								
Measured at continuous monitor:												
Urban background	0.21	0.19	0.18	0.31	1.0							
Rural background	0.24	0.18	0.27	0.76	0.59	1.0						
Street level	0.13	0.005	0.01	0.004	0.006	0.02	1.0					
Global radiation	0.27	0.10	0.0009	0.24	0.13	0.22	0.06	1.0				
Air pressure	0.04	0.08	0.0004	0.0009	0.07	0.002	0.09	0.0009	1.0			
Precipitation	0.06	0.03	0.09	0.14	0.08	0.14	0.09	0.03	0.04	1.0		
Temperature	0.09	0.005	0.18	0.05	0.004	0.03	0.16	0.56	0.05	0.008	1.0	
Wind speed	0.07	0.008	0.06	0.001	0.03	0.01	0.17	0.12	0.07	0.03	0.12	1.0
**C) BC averaged over 1 year**												
	**Personal measurements**		**Modelled for home address**	**Modelled for work address**								
Personal measurements:	Parent on a leave	Working parent										
Parent on a leave	1.0											
Working parent	0.69	1.0										
Modelled for home address	0.20	0.09	1.0									
Modelled for work address	NA	0.13	0.08	1.0								

For the 1-week average personal BC levels the pattern was similar (Table 1B). Here also other weather factors like global radiation and precipitation showed some correlation. When the observed personal BC levels were standardized to reflect annual averages (using the urban background station as reference)20% and 9% of their variance was explained by the modelled outdoor levels at home (R^2^ = 0.20 and 0.09, respectively) and 13% by modelled outdoor levels at work (R^2^ = 0.13; Table 1C).

### Predictors of personal BC exposure

In a restricted dataset of 24 h observations of personal BC exposure (excluding records with missing observations in urban background, *n* = 160), and disregarding the repeated sampling structure of the data, multiple regression analyses were performed in two sets, using: (1 Fixed monitoring data with and without weather variables, /92) Time-resolved modelling data with and without weather variables.

Using monitoring data only (RB, UB-RB, ST-UB), the best model explained 23% of the variability (R^2^adj) for both parents combined (Table 2), based on monitors for RB and UB. Including data from ST did not improve the model, and of the weather variables air pressure made a very slight improvement. When analyzing working and on-leave parents separately, the variability for the working parent was slightly better explained. Sensitivity analysis based on the complete dataset, ie including 11 unreasonably high 24 h average BC values (Supplementary Table S3) showed comparable results to those obtained in our main analysis presented in Table 2.

**Table 2:** Personal 24 h average black carbon exposure (ng/m^3^) with monitoring data, air pressure (standardized to mean 0 and SD 1) and season (Cold vs Warm). Multiple regression coefficients based on 7 day observations (*N* = 59).

Parent	Rural background (RB)	Urban background (UB)-RB	Street (ST)-UB	Air pressure	Season	Intercept	n	R^2^adj
Both	0.67 (0.53–0.81)	0.31 (0.15–0.47)				204 (162–247)	305	0.23
Both	0.64 (0.50–0.78)	0.37 (0.19–0.55)	–0.05 (–0.11 to 0.009)			238 (184–292)	300	0.22
Both	0.63 (0.49–0.77)	0.31 (0.15–0.48)		22.3 (1.55–43.1)		212 (169–255)	305	0.24
Both	0.71 (0.56–0.87)	0.30 (0.14–0.47)			–56 (–147 to 35.0)	202 (159–245)	305	0.23
On-leave	0.67 (0.46–0.89)	0.32 (0.07–0.56)		16.9 (–13.3 to 47.0)		206 (142–270)	155	0.21
Working	0.58 (0.39–0.77)	0.31 (0.09–0.53)		28.8 (–0.29 to 57.8)		219 (160–278)	150	0.25

If instead using time-resolved modelled outdoor BC levels at home and at work, the best model for the working parent used modelled data at home and work, and air pressure, explaining 34% of the variability (Supplementary Table S4A). For the on-leave parent the best model explained 20% of the variability. In a sensitivity analysis based on the complete dataset, ie including 11 unreasonably high 24 h average BC values, we observed same degree of explained variability of the best model for the on-leave parent, ie 20%, while for the working parent corresponding model explained 23% of the variability (Supplementary Table S4B).

For the parent on leave the self-administered diary information did not seem to relate to personal 24 h BC, while for the working parent more time outdoors increased personal BC levels and more time at work decreased these levels, possibly because of low BC levels indoors at the workplaces. When incorporating also these variables in the models with air pressure, up to 39% of personal BC variability could be explained (Supplementary Table S4A).

In an alternative approach we regressed the intra-person BC variability (by subtracting the personal weekly mean from each 24 h mean observation) on the corresponding within-week variation in stationary monitoring data or in modelled data for home and work (Supplementary Table S5). In these multiple regressions up to 24% of the personal within-week variability could be explained for the parents combined and up to 37% for the working parent.

When summarized per week, the explanatory power in multiple regression using stationary monitoring data and weather data remained below 20% (data not shown), but with modelled data and season, the on-leave and working parent’s variability could be explained at 39% and 45%, respectively (Supplementary Table S6). When using modelled BC levels both at home and at work (working parent only), the estimated annual personal BC levels could be explained at 33% for both parents (Table 3).

**Table 3:** Estimated personal yearly average black carbon exposure (ng/m^3^) with modelled outdoor levels at home and work. Multiple regression coefficients (*N* = 23).

Parent	Modelled at home	Modelled at work	Intercept	*n*	R^2^adj
Yearly average BC levels					
On-leave	0.38 (0.15–0.62)		198 (62.2–334)	23	0.33
Working	0.40 (0.12–0.69)	0.17 (-0.39-0.73)	127 (–114 to 368)	20	0.33

## Discussion

Our main findings are that personal BC exposure for inner-city dwellers to a certain extent followed the daily patterns at fixed monitoring stations, that personal exposure on average corresponded well both to urban background measurements and to levels modelled at place of residency, but that monitoring data or time-resolved dispersion modelling data only explained the variability in 24 h exposure to less than 35% a working parent and even less for a parent on parental leave. Neither weather data nor time-activity data made any important improvements. For the weekly average BC exposure, the situation was somewhat better when using modelled BC data, reaching up to 45% explanation for a working parent. When using the personal weekly average to estimate long-term exposure, this was explained at 33% by dispersion modelling data.

### Comparison with previous studies

Most other studies of personal BC were focused on the role of different microenvironments for individual exposure and inhaled dose, as a basis for policy [5, 6]. We found similar average diurnal patterns with low night-time levels, and for working parents distinct peaks corresponding to commuting, but somewhat less distinct diurnal patterns for the parents on leave. Interestingly the parent groups showed nearly identical patterns on Saturdays, perhaps reflecting joint activities, but not on Sundays, possibly reflecting an exchange of duties between the parents. However, we had no additional information about specific activities from the time-activity diaries and hence we can only speculate on this matter.

It can be noted that the personal BC exposure levels in our study were lower than in most other reports, even in comparison with data from e.g. Birmingham [19], Brisbane [20] or Paris [21], reflecting a comparatively well controlled outdoor environment, and the absence of major BC indoor sources for our study population.

In the perspective of estimating population exposure, it can be noted that in our study the BC personal exposure levels were on weekly average only about 30% higher than the corresponding UB, indicating that UB might be used for estimating average exposure to ambient BC for inner-city dwellers. This is in contrast with other studies that concluded that ambient monitoring did not provide adequate estimates of average population exposure [21, 22]. The reason for this discrepancy might be that our study persons lived close to the UB station, and also reported no smoking or use of open fires. The alternative in this study, using time- and space-resolved dispersion modelling produced even closer estimates, with an average about 10% overestimation of the personal exposure over a one-week period, and might also be better suited for assessing average population exposure in larger areas.

In the perspective of a time-series study using 24 h urban background monitoring data, our results are less promising, as only up to 25% of the temporal variability in personal exposure could be explained by the variability at urban and rural background stations, and that including season or weather data only provided marginal improvements, in contrast to other studies [23]. Data from the street-site monitor did not seem to contribute. Time-series studies of health effects from BC in Stockholm or similar cities are thus to be expected to suffer from substantial bias towards the null.

In total, 24 h dispersion modelling estimates for the outdoor levels at the home address gave similar results, explaining up to 26% of the variability in personal exposure, less than in other similar studies [23, 24]. One reason for this might be the lower general levels of BC in Stockholm. For the working parents up to 34% could be explained by adding dispersion modelling estimates for the work address, and air pressure, indicating that for a working population some exposure assessment precision might be gained using dispersion modelling for both home and work locations. This might however not be that relevant for studies of mortality and other health effects that are less common within working populations.

Information on time-activity patterns from the self-administered diary did not help to explain the variability in 24 h personal exposure levels, in contrast to other reports [5, 6, 21–23, 25, 26], with the only exception of time spent at work, probably because most workplaces in the region have mechanical ventilation systems with filters decreasing indoor levels of BC, while most inner-city homes have no treatment of inlet air. The indoor/outdoor BC ratio (I/O) for the workplaces was not measured in this study, but the in the central hours of the workday low levels were recorded. We have previously reported that the I/O for the homes in this study population was on average 79% [8].

While in the long-term perspective, differences in health effects between pollutants with similar spatial spread are difficult to discern, short-term studies of acute effects may shed some light, as different pollutants may show different temporal patterns But when the relation between the temporal air pollution metric used—often the level in urban background—and personal exposure, differs between pollutants, also the degree of bias will differ, invalidating a direct comparison between pollutants [27]. Our finding of a low temporal correlation between UB and personal exposure may thus indicate a relative handicap for BC in short-term studies of multiple pollutants.

### Strengths, limitations, and further research

Our study has the merit of personal BC measurements being performed in a low-level city environment and using not only monitoring station data but also time-resolved spatial estimates both at home and work addresses. One limitation is that the study area was quite small why our results may not be readily applied to large city areas. The summer period was not well covered, why we had limited power to address seasonal differences. Our self-administered diary data were on purpose quite simple in order to mimic a possible large-scale study. Further, different instruments (AE33 versus AE51) used at fixed monitoring sites and for personal exposure could partly contribute to decrease explained variance of the personal exposure. But as shown by e g Alas et al., the AE33 and AE51 are highly correlated if the filter loading effect is under control [28]. Another noteworthy limitation of our analyses based on variable selection approach is that it ignores repeated sampling within individuals, which renders the estimated standard errors and confidence intervals.

Although the majority of people spend most of their time indoors, the focus of both research and policy on air pollution has been on outdoor levels. While this in practice might be the only possibility, it must be acknowledged that the imperfect relation between outdoor levels and personal exposure is an important source of bias. Not only does it tend decrease statistical power in studies of health effects in general, it may also distort the view of the relative importance of different pollutants. We thus believe that quantifying this bias is of great importance both for science and for policy.

In conclusion, one-week continuous personal measurements performed by inner city adult Stockholmers showed exposure levels in the order of 400 ng black carbon/m^3^, with distinct diurnal and weekly patterns. Average exposure levels were similar to data from routine urban background monitoring or dispersion model estimates, indicating that *long-term* population exposure and related health effects may be estimated based on such data. The variability of 24 h exposure levels could however only be explained at about 35%, using routine monitoring or dispersion modelling. Any short-term health effects studies using such exposure data are likely to lack power and to be subject to bias.

### Supplementary information

Appendix

## Data Availability

The data that support the findings of this study are not publicly available because they include personally identifiable information that could compromise research participant privacy/consent.
